# Understanding the management of pediatric spondylodiscitis based on existing literature; a systematic review

**DOI:** 10.1186/s12887-023-04395-2

**Published:** 2023-11-18

**Authors:** Narges Lashkarbolouk, Mahdi Mazandarani, Brice Ilharreborde, Mohammad Hossein Nabian

**Affiliations:** 1 Endocrinology and Metabolism Research Institute, Tehran University of Medical Sciences, Tehran, Iran; 2 Golestan University of Medical Sciences, Gorgan, Iran; 3 Department of Pediatric Orthopedic Surgery, Robert Debré Hospital, AP-HP, Paris Diderot University, Paris, France; 4 Center for Orthopedic Trans- Disciplinary Applied Research (COTAR) Institute, School of Medicine, Tehran University of Medical Sciences, Tehran, Iran

**Keywords:** Discitis, Spondylodiscitis, Children, Spinal disease, Intervertebral disk

## Abstract

**Background:**

Spondylodiscitis (SD), a rare disease in children, poses diagnostic challenges due to non-specific presenting symptoms, scarcity in incidence, and difficulty expressing pain in non-verbal children.

**Method:**

A comprehensive search was conducted on three databases, including PubMed/Medline, Web of Science, and Scopus until March 2023. The inclusion criteria were studies that investigated the clinical characteristics, treatment, and complications of children’s spondylodiscitis. Full text of cross-sectional and cohort studies were added. The quality assessment of cohort studies was conducted using the Newcastle-Ottawa Quality Assessment Scale. The search, screening, and data extraction were performed by two researchers independently.

**Result:**

Clinical manifestations of discitis in children are nonspecific, such as back pain, fever, reduced ability or inability to walk or sit, limping, and reduced range of movements. The mean delay in the time of diagnosis was 4.8 weeks. The most affected site of all the studies was the lumbar spine. 94% of studies reported increased inflammatory markers such as white blood cell count, C-reactive protein, and erythrocyte sedimentation rate. Less than 30% of patients had positive blood cultures and biopsy findings. The most common microbiological results (64%) were *Staphylococcus Aureus* and *Kingella kingae*. In radiographic evaluation, intervertebral disk narrowing, lumbar lordosis reduction, loss of disk height, and destruction of the vertebral body have been reported. In all studies, antibiotic therapy was initiated; in 52% immobilization was employed, and 29% of studies reported surgery was performed, and the follow-up period differed from 1.5 months to 156 months. 94% of studies reported complications such as vertebral body destruction, back pain, kyphosis, reduced range of movement, scoliosis, and neurological complications.

**Conclusion:**

Spondylodiscitis is an uncommon, heterogeneous, multifactorial disease with resulting difficult and delayed diagnosis. Due to its morbidity, it is essential to investigate children with refusal to walk, gait disturbances, or back pain, particularly when associated with elevated inflammatory markers.

**Supplementary Information:**

The online version contains supplementary material available at 10.1186/s12887-023-04395-2.

## Introduction

Childhood spondylodiscitis is a rare disease that represents infection or inflammation of the intervertebral disc space or vertebral endplate. Childhood discitis incidence is about 1:250,000, corresponding to about 2–4% of infectious bone diseases, and the average age at diagnosis is between two and eight years. To this day, the definite etiology and pathophysiology of spondylodiscitis are unknown due to the unique anatomical features of the pediatric spine. However, from an etiological perspective, spinal infections can be divided into pyogenic and non-pyogenic (granulomatous and parasitic) infections [1–3].

The causes of spinal infections can be hematogenous (arterial or venous—via the Batson plexus), direct external inoculation (implantation after trauma, surgery, punctures, and epidural procedures), and contiguous spread to the bone from adjacent tissues. Hematogenous spread via the arterial system is the most common source of infection, as the arterial system strongly supplies the vertebral body, and the distribution of the blood vessels of the spine is related to age. Therefore, the infection site may differ at different ages, e.g., otitis media, urinary tract infections, or lung infections [4–8].

Clinical manifestations mainly include nonspecific, variable, and not always limited to spinal symptoms, such as enigmatic abdominal or back pain exacerbated by movement and worsening at night, fever, limping or refusal to bear weight, abnormal posture, inability to sit, and claudication. As stated in the studies, these nonspecific manifestations can delay diagnosis by up to three months in 50% of patients. The assessment of clinical signs in children under one year of age due to their inability to express pain or symptoms has also made timely diagnosis difficult. In the past medical history, parents often report preceding viral or bacterial infection, history of trauma, or history of surgery [6–10].

Typical findings on physical examination are fever, inability to walk or abnormal gait, inability to sit, limited hip movement, local tenderness of the spine, paraspinal muscle spasms, limited spinal mobility, hamstring muscle tightness, decreased lumbar lordosis, a positive straight leg raising test (SLR), limb weakness, decreased tone, absent reflexes, and a positive log-roll test (for assessing hip pain) [11–15].

The diagnosis of spondylodiscitis can be based on a combination of clinical signs, laboratory tests, and imaging. The disease course is often chronic, and the absence of specific symptoms often delays the diagnosis. This delay in diagnosis increases morbidity and mortality. Although any level of the spine can be affected, in most cases (75% of patients), the lumbar spine or lumbosacral region is mainly affected, especially in children under five [5, 7, 16–18].

In the laboratory investigation, the most common features are elevated erythrocyte sedimentation rate (ESR), C-reactive protein (CRP) level, and leukocytosis. Conversely, the results of blood culture and fine needle aspiration (FNA) sample culture tests varied in different studies [17–20].

Radiological evaluation usually starts with plain radiographs. X-rays have very low sensitivity and specificity, and after 2–3 weeks, a simple X-ray shows isolated intervertebral disc space narrowing. Then, within 3–4 weeks, irregularities in the vertebral plate, tooth erosion of adjacent vertebral endplates, and scaling of the superior and inferior vertebral bodies can be detected in plain X-ray evaluation. For further evaluation, computed tomography (CT) scans, magnetic resonance imaging (MRI), and technetium Tc 99 m (99mTc) may be used. MRI is the gold standard method, with higher sensitivity (96%) and specificity (94%) than nuclear studies or CT for detecting discitis early. The MRI allows visualization of the disk, neural tissues, surrounding soft tissues, and pathophysiologic changes in the vertebral body [5–16].

Differential diagnoses of discitis can include Scheuermann’s kyphosis, Schmorl’s nodes, destructive pyogenic osteomyelitis, septic arthritis, tuberculosis infection, brucellosis, various metastatic tumors, osteoid osteoma, osteoblastoma, neuroblastoma. Compared to spondylodiscitis, these diseases involve the vertebral body and endplates and often occur at multiple levels throughout the spine, mostly sparing the disc [17–24].

Spondylodiscitis in children can result in serious complications, some lifelong, imposing enormous costs on the patients and the healthcare system thus increasing the burden of disease. To improve the prognosis clinicians need to be knowledgeable about the signs and symptoms of spondylodiscitis thus preventing untoward delay in diagnosis. To better understand the impact of delayed diagnosis some studies have followed patients beyond the completion of treatment [6–9, 11–22].

The goal of management is to eliminate the infection and restore and preserve the function and structure of the spine, relieving the pain. The treatment of this disease includes antibiotic therapy, immobilization, and surgery if indicated [19–22].

Unlike adults, discitis in children is usually benign due to better blood supply and should recover without complications. Nevertheless, delay in diagnosis can lead to persistent unpleasant side effects, drug resistance, and invasive treatment such as surgery in patients. The study by Kayser R. et al. (2005) reported that the mean delay of SD diagnosis was 14 weeks, and these cases faced complications such as fusion of the vertebra, restricted spinal movement, local kyphosis, and residual defects [15]. The study by Afshari et al. (2019) reported that pediatric discitis has a more nonspecific clinical presentation than adults, making diagnosis more challenging and potentially having a negative evolution. Discitis diagnosis heavily relies on a high index of suspicion to prompt investigations, particularly in cases with no overt clinical symptoms of infection (fever or seizures). Also, they mentioned that although most cases are successfully treated with antibiotics, significant complications are reported in non-verbal babies or children due to their inability to express themselves [6]. As mentioned earlier, a lack of standard treatment guidelines is notable due to this disease’s unclear etiology and pathophysiology, unspecified clinical manifestations, and diverse laboratory or radiographic evaluation results. This study aimed to design a comprehensive, systematic review of spondylodiscitis management in children.

## Methods

This systematic review investigated the clinical characteristics, treatment, and complications of children’s spondylodiscitis. The guidelines of Preferred Reporting Items for Systematic Review and Meta-analysis (PRISMA) for developing the current systematic review followed [25].

### Search strategy

A systematic literature search was performed on three electronic databases, including PubMed/Medline, Web of Science, and Scopus, and we used standard keywords until March 2023.

The following search string was: “discitis” OR “diskitis” OR “Spondylodiscitis” OR “Spondylodiskitis” OR “intervertebral disc disease” OR “disc disease” OR “disk disease” AND “children” OR “pediatric” OR “childhood” OR “child.“

### Study selection

Two researchers independently screened all studies for eligibility based on title, abstract, and full text. In the third author’s opinion, any disagreements were discussed and resolved.

The inclusion criteria:

1. Studies investigated the impacts of children’s discitis/spondylodiscitis.
2. The population of interest was children.
3. Type of study: Observational and clinical trials studies.
4. Studies that their full-text were available in English.

The exclusion criteria:

1. Studies which not reported the impacts of children’s discitis/spondylodiscitis
2. Reviews, commentaries, case studies, and letters

Duplicated studies were removed, and disagreements between authors at the screening stage were discussed and resolved.

### Data collection process

Data regarding the impacts of children’s discitis was retrieved.

The extracted data included:

1. General characteristics of the included studies (first author name, year of publication, country, study population, study setting).
2. Methodological characteristics (study design, sample size, and quality score).
3. Clinical characteristics (clinical presentation, site of infection, delay in the time of diagnosis, laboratory findings, microbiological findings, radiographic findings), treatment, and complications.

### Quality assessment

All included studies were reviewed for quality assessment scores using the Newcastle-Ottawa Quality Assessment Scale (NOS) for cross-sectional and cohort studies [26, 27]. Two independent investigators assessed the quality of included studies. In case of any disagreement, the senior author’s opinion was resolved.

This scale consists of evaluating the methodological quality of the studies in eight items for cohort studies and seven items for cross-sectional studies that contain three categories: Selection of participants (maximum four scores), comparability of subjects (maximum two scores), and assessment of outcome (maximum three scores).

According to quality assessment scales, after calculating scores for cross-sectional studies, we classified 9 and 10 points as “very good,“ 7 and 8 points as “good,“ 5 and 6 as “satisfactory,“ and 0 to 4 as “unsatisfactory.“ For cohort studies, “good quality” studies define as if a study achieves 3 or 4 points in the selection part, AND 1 or 2 points in the comparability part, AND 2 or 3 points in the outcome part. “fair quality” studies defined as, if a study achieves two scores in the selection part, AND 1 or 2 scores in the comparability part, AND 2 or 3 points in the outcome part. In addition, if a study gets 0 or 1 in the selection part OR 0 score in the comparability part OR 0 or 1 score in the outcome part, it is considered “poor quality” **(Supplementary Tables 1,2)**.

### Statistical analysis

Due to heterogeneity between studies in terms of outcomes, outcome tool assessment, study design, and study setting, the results were analysed qualitatively.

## Results

### Search results and study selection

**Fig. 1:**
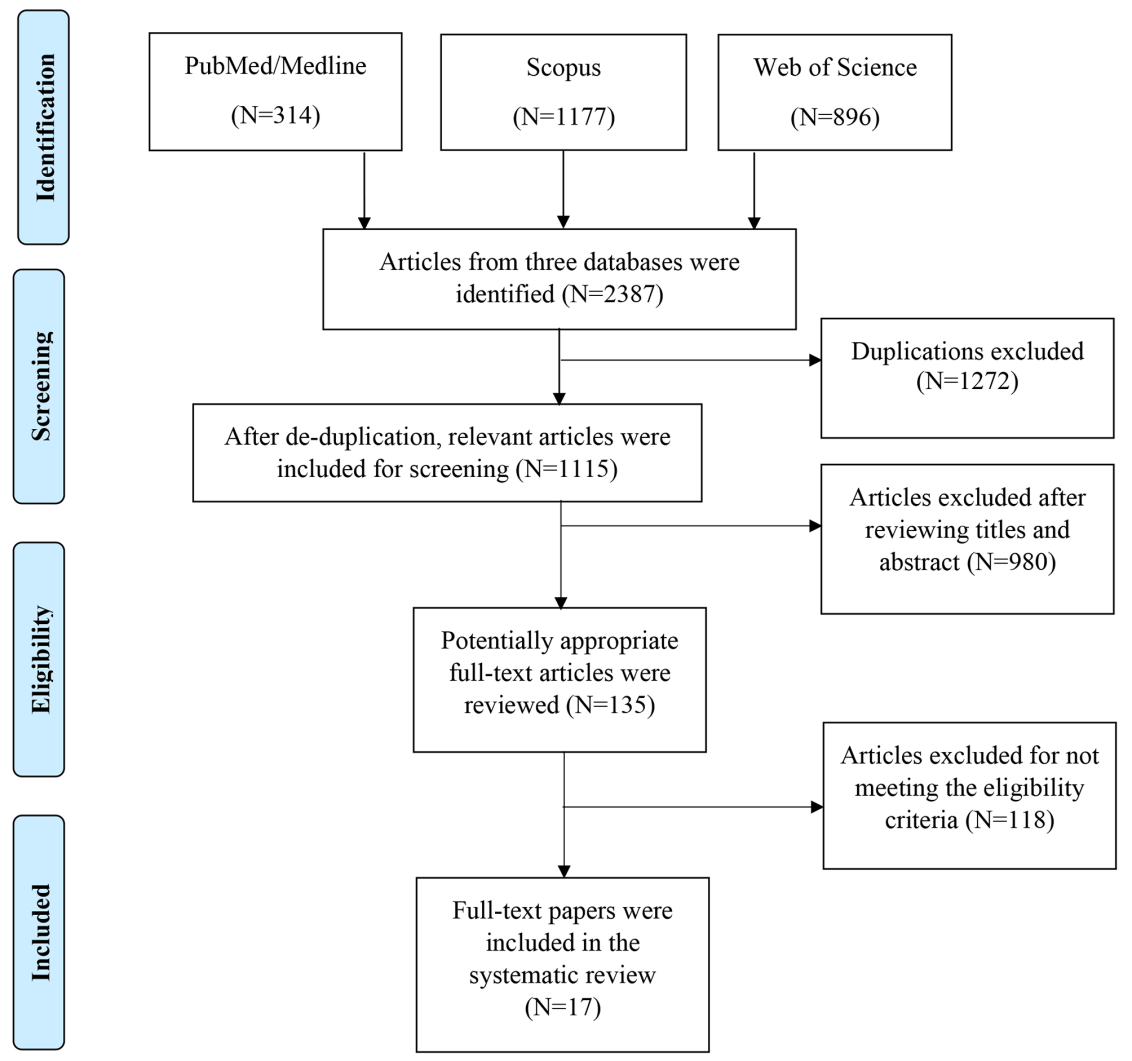
Flow Chart of Study Selection Process

In Fig. 1, the PRISMA flowchart is shown for study selection. In search of the three databases, 2387 studies were identified (PubMed = 314, Scopus = 1177, Web of Science = 896). After removing 1272 duplicate documents, 1115 studies remained. After reviewing titles and abstracts,980 studies were disqualified. One hundred thirty-five studies were reviewed in full text, and studies that did not specifically investigate the impacts of children’s discitis were excluded. Finally, for this systematic review, 17 studies were selected.

### Study characteristics

Table 1 presents a summary of the 17 studies for this systematic review. Sixteen studies were retrospective, and one was cross-sectional. All studies were published between 2001 and 2022. Studies were investigated in the different continents such as Europe (Switzerland = 2, Scotland = 1, Spain = 1, Italy = 3, France = 1, Germany = 3, England = 4) [6–12, 14–21], Asia (South Korea = 1) [13], and Africa (Benin = 1) [22]. The total number of children for this systematic review was 418.

**Table 1:** General characteristics of included studies

First author,(Year)	Country	Study setting	Study design	Study population	Sample size	Quality score
Yagdiran A.,et al.2022	Germany	University Hospital of Cologne, Cologne	Retrospective, Cohort study	Children Mean age: 5 years 30% girls 70% boys	N = 10	Good quality
Ferri I.,et al.2021	Italy	Meyer children’s University Hospital, Florence	Retrospective, Cohort study	Children Mean age: 9.3 years 42.8% girls 57.2% boys	N = 21	Good quality
Musso P.,et al.2021	Italy	Meyer children’s University Hospital, Florence	Retrospective, Cohort study	Children Mean age: 5 years 45.4% girls 54.6% boys	N = 22	Good quality
Roversi.M.,et al.2021	Italy	Bambino children’s Hospital, Rome	Retrospective, Cohort study	Children Mean age: 3.8 years 47.9% girls 52.1% boys	N = 48	Good quality
Afshari F.,et al.2019	England	Birmingham children’s Hospital, Birmingham	Retrospective, Cohort study	Children Mean age: 4.3 years 52.1% girls 47.9% boys	N = 23	Good quality
Dayer R.,et al. 2018	Switzerland	University Hospital of Geneva, Geneva	Retrospective, Cohort study	Children Mean age: 3.4 years 34.9% girls 65.1% boys	N = 103	Poor quality
Kang H.,et al.2016	South Korea	Seoul national University children’s Hospital, Seoul	Retrospective, Cohort study	Children Mean age: 13.8 years 40% girls 60% boys	N = 25	Good quality
Ceroni D.,et al.2013	Switzerland	Geneva children’s Hospital, Geneva	Retrospective, Cohort study	Children Mean age: 2.8 years 40% girls 60% boys	N = 10	Good quality
Zomalheto Z.,et al.2013	Benin	National Hospital University hubert Koutoukou Maga, Cotonou	Cross-sectional study	Children Mean age: N.A. 37.9% girls 62.1% boys	N = 29	Good quality
Spencer S.,et al.2012	Scotland	Children’s Hospital of Scotland, Glasgow	Retrospective, Cohort study	Children Mean age: 1.8 years 75% girls 25% boys	N = 12	Good quality
Chandrasenan J.,et al. 2011	England	Royal Derby Hospital, Derby	Retrospective, Cohort study	Children Mean age: 3.3 years 56.2% girls 43.8% boys	N = 16	Good quality
Miranda.I.,et al.2008	Spain	Department of orthopaedic surgery and traumatology, Valencia	Retrospective, Cohort study	Children Mean age: 1.6 years 30% girls 70% boys	N = 10	Good quality
Waizy H.,et al.2007	Germany	Pediatric department of the Heinrich-Heine University, Düsseldorf	Retrospective, Cohort study	Children Mean age: 1.9 years 50% girls 50% boys	N = 6	Good quality
Kayser R.,et al.2005	Germany	University orthopedic Hospital, Magdeburg	Retrospective, Cohort study	Children Mean age: 6.1 years 68% girls 32% boys	N = 25	Good quality
Karabouta Z.,et al.2005	England	Bristol royal Hospital, Birmingham	Retrospective, Cohort study	Children Mean age: 1.1 years 60% girls 40% boys	N = 5	Good quality
Garron E.,et al. 2002	France	Children’s Hospital of Timone, Marseille	Retrospective, Cohort study	Children Mean age: 4.6 years 45.2% girls 54.8% boys	N = 42	Good quality
Brown.R.,et al.2001	England	Children Hospital of London, London	Retrospective, Cohort study	Children Mean age: 1.5 years 63.6% girls 36.4% boys	N = 11	Good quality

### Quality of studies

For cross-sectional and cohort studies, by using quality assessment criteria of the Newcastle-Ottawa Quality Assessment Scale, fifteen retrospective cohort studies (90%) had good quality [6–9, 11–21], one retrospective cohort study (5%) had poor quality [10], and one cross-sectional study (5%) had good quality [22]. The quality of all studies is shown in Table 1.

### Demographic characteristics, clinical features, site of involvement

**In** Table 2, the age at the presentation of childhood spondylodiscitis was highly variable, ranging from 24 days to 17.5 years. After analyzing 418 spondylodiscitis cases, 55% were male, and 45% were female, with a mean age of 4.3. One study did not report the mean age of children and just said that the teenager group was the most represented age group (65.5%), then the grade schoolers (24.1%), and the toddlers (10.4%) [22].

**Table 2:** Clinical and etiological characteristics of included studies

First author, (Year)	Clinical presentation	Site of involvement	Delay in the time of diagnosis	Laboratory findings	Microbiological findings	Radiographic findings	Follow up and Complications	Treatment
Yagdiran A.,et al.2022	Reduced ability or inability to walk or sit (70%), limp (60%), back pain (50%), abdominal pain (10%), fever (10%)	Thoracic spine (10%), lumbar spine (90%)	9.2 weeks	CRP elevation (60%), ESR elevation (50%), anemia (10%)	Negative blood cultures (100%), Biopsy: *M.tuberculosis* (10%), *MSSA* (10%)	X-rays: narrowing of intervertebral disk (50%) MRI: reduced disk height, disk hypointensity on T1, and disk hyperintensity on T2 (100%)	1.5 months follow-up: destruction of vertebral body (70%), psoas abscess (40%), paraspinal abscess (30%)	Antibiotics (100%) for 5.2 weeks, surgical intervention (20%)
Ferri I.,et al.2021	Reduced range of movement (66.6%), back pain (49.8%), limp (19%), fever (33.3%), reduced ability or inability to walk or sit (14.2%), neurological sign (4.7%), torticollis (4.7%),	Cervical spine (10%), thoracic spine (20%), lumbar spine (60%), sacral (10%)	1.4 weeks	ESR elevation (85.7%), leukocytosis (33.3%), CRP elevation (19%)	Positive blood culture and biopsy: *S.aureus* (28.6%)	X-ray: reduction of lumbar lordosis, loss of disk height (22.4%) MRI: disk hypo intensity on T1, and disk hyper intensity on T2 (100%), subperiosteally and muscular abscess (14.3%)	12 months follow-up: back pain (25%), kyphosis (10%)	Antibiotics (100%) for 4weeks, immobilization (bracing) (57.1%), surgical intervention (4.75%)
Musso P.,et al.2021	Back pain (72.7%), reduced range of movement (72.7%), fever (31.8%), swelling (4.5%)	Cervical spine (13.8%), thoracic spine (13.8%), lumbosacral spine (72.4%)	1.7 weeks	ESR elevation (100%), CRP elevation (77.8%), leukocytosis (19.5%)	Positive blood culture: *S.aureus* (13.6%), *E.coli* (4.5%), *S.agalactiae* (4.5%)	X-rays: narrowing of intervertebral disk (100%) MRI and CT scan: Subperiosteally abscess (4.5%), muscular abscess (9%)	Follow-up: Reduced range of movement (5%)	Antibiotics (100%) for 7weeks, surgical intervention (4.5 %)
Roversi.M.,et al.2021	Back pain (96%), fever (46%) limp (10%)	Cervical spine (6.5%), thoracic spine (24.5%), lumbar spine (44%), lumbosacral (25%)	3weeks	ESR elevation (100%), CRP elevation (in few cases)	Positive blood culture: *S.aureus* (10.4%), *M.tuberculosis* (14.3%), *K. kingae* (8.3%)	X-rays: narrowing of intervertebral disk (96%) MRI and CT scan: Involvement of adjacent vertebral endplate (33%), soft tissue abscess (33%), Cellulitis or myositis (15%)	12.5 months follow-up: kyphosis (10.4%), scoliosis (4.1%), gibbus (2%), rigidity (2%)	Antibiotics (100%) for 6 weeks, immobilization (cast braces) for16weeks (100%)
Afshari F.,et al.2019	reduced ability or inability to walk or sit (57%), back pain (52%), fever (14%), limp (10%)	Cervical spine (4.7%), thoracic spine (4.7%), lumbar spine (90.6%)	6 weeks	ESR elevation (66.6%), CRP elevation (38%), leucocytosis (9.5%)	Positive blood culture: *S.aureus* (4.75%), mixed *E. coli* and *M.morganii* (4.75%), Biopsy: *S.aureus* (one case)	X-rays: narrowing intervertebral disc (100%) MRI: abnormal disc signal (100%), epidural spinal collection (4.76%)	20 months follow- up: back pain (19%)	Antibiotic (100%) for 9.3 weeks, no surgery management
Dayer R.,et al. 2018	Fever (36%), reduced general condition (36%)	Cervical spine (6%), thoracic spine (18%), lumbar spine (70%), sacral (6%)	4weeks	ESR elevation (86%), thrombocytosis (63%), CRP elevation (58%), leukocytosis (41%)	Positive blood culture: *S.aureus* (6%), *K. kingae* (2%) Biopsy (7.7%): *S.aureus*, *K. kingae*, *L.lactis*	X-rays: narrowing intervertebral disk (100%) MRI: low signal intensity on T1, a loss of definition of the endplate and of the adjacent vertebral bodies, and high signal intensity on T2 (100%), Vertebral osteomyelitis (29%)	N.A.	Antibiotic (100%), no surgical management
Kang H.,et al.2016	Back pain (68%), fever (52%), reduced ability or inability to walk or sit (62.5%), abdominal pain (20%), neurological sign (12%)	Cervical spine (8%), thoracic spine (16%), lumbar spine (72%) sacral (4%)	6.4 weeks	ESR elevation, CRP elevation, and leukocytosis (in most cases)	Positive blood culture and biopsies: *S.aureus* (40%), *M.tuberculosis* (32%), *E.coli* (8%), *S.pneumonia* (4%), *S.typhi* (4%)	X-rays: destruction of vertebral body (80%), disk involvement (100%) MRI and CT scan: disk involvement (100%), involvement of adjacent vertebral body (76%), paravertebral abscess (23.5%)	60 months follow-up: kyphosis (8%), scoliosis (8%), back pain (4%)	Antibiotic (100%) for 6-61weeks, surgical intervention (48%)
Ceroni D.,et al.2013	Fever (90%)	Lumbar spine (100%)	4.2 weeks	ESR elevation (90%), CRP elevation (60%) thrombocytosis (70%)	Negative blood cultures (100%),	MRI: abnormal disc signal (100%), abnormal signal in adjacent vertebral body with bone abscess (60%), epidural abscess (20%)	48 months follow-up: no evidence of complications	Antibiotics (100%), no surgical management
Zomalheto Z.,et al.2013	Back pain (72.4%), weight loss (72.4%), fever (58.6%), anorexia (41.3%), neurological sign (7%)	Thoracic spine (27.6%), lumbar spine (72.4%)	6.5 weeks	ESR elevation (100%), CRP elevation (100%), leukocytosis (100%)	Positive blood culture: *M.tuberculosis* (72.4%), *S.aureus* (4.76%), *E.coli* (14.28%), *K.pneumonia* (4.76%), *Salmonella spp*. (4.76%)	N.A.	10 months follow-up: kyphosis (31%), neurological complications (31%)	Antibiotics (100%), and immobilization (100%) for 12weeks, no surgical management
Spencer S.,et al.2012	Limp (70%), back pain (70%), abdominal pain (20%)	Lumbar spine (100%)	3.1weeks	ESR elevation (100%), CRP elevation (16%), leukocytosis (16%)	Positive blood culture: Gram positive Cocci(17%)	X-rays: narrowing of intervertebral disc height (100%) Bone scan: increase uptake at effected disk (100%) scan: demonstrating intervertebral disc involvement (100%), abscess (16%), epidural collection (8%)	13.3 months follow up: no evidence of complications	Antibiotics (100%) for 6 weeks, no surgical management
Chandrasenan J.,et al. 2011	Reduced ability or inability to walk or sit (56%), fever (37.5%), back pain (31.5%), reduced range of movement (31%), reduced general condition (12.5%), abdominal pain (6.25%)	Thoracic spine (37.5%), lumbar spine (62.5%)	2.5weeks	ESR elevation (87.5%), CRP elevation (50%), leukocytosis (31.25%)	Positive blood culture: *S.aureus* (31.2%), *S.epidermis* (6.2%), *S.pneumonia* (6.2%)	X-rays: narrowing of intervertebral space (100%), sclerosis and fusion of the vertebra or ankyloses (50%), Bone scan: increase uptake at effected disk and adjacent vertebral bodies (25%) MRI: abnormal disc signal (75%)	24 months follow-up: restrictions in spinal movements ( 20%), disk degeneration (37.5%)	Antibiotics (87.5%), immobilization (bracing) (37.5%), no surgical management
Miranda.I.,et al.2008	Reduced ability or inability to walk or sit (70%),back pain (80%), fever (30%)	Cervical spine (10%), lumbar spine (90%)	3.7 ± 0.6 weeks	ESR elevation (100%), CRP elevation (80%), leukocytosis (80%)	N.A.	X-rays: reduction of the intervertebral space, and irregularity of the vertebral (40%) MRI: abnormal disc signal (100%), paravertebral abscess (60%)	156 months follow-up: sclerosis, osteophytes, and reduction of intervertebral space (80%)	Antibiotics (100%) for 11weeks, immobilization for 21.2 weeks, no surgical management
Waizy H.,et al.2007	Limp, and reduced ability or inability to walk or sit (100%), back pain (83%), abdominal pain (16%), fever (16%)	Thoracic spine (16%), thoracolumbar spine (33%), lumbar spine (50%)	3.5weeks	ESR elevation (100%)	Negative blood cultures (100%)	X-rays: decreased height of disk space, erosions of adjacent vertebral endplate (100%) MRI: abnormal disc signal (100%)	31 months follow-up: sclerotic vertebral endplates or partial fusion (few cases)	Antibiotics (100%) for 3 weeks, immobilization (100%) (cast or corset) for 40 weeks, no surgical management
Kayser R.,et al.2005	Fever (100%), reduced range of movement (88%), back pain (76%), reduced ability or inability to walk or sit (44%), reduced general condition (28%)	Cervical spine (4%), thoracic spine (36%), lumbar spine (60%)	14 weeks	ESR elevation (84%)	Negative blood cultures (100%)	X-rays: disc space narrowing (100%), destruction of adjacent vertebral bodies (48%), isolated disc involvement (52%) CT scan and bone scan: inflammatory changes in the affected regions (16%), abscess (4%)	120 months follow up: fibrous ankylosis and high-grade narrowing of the intervertebral disc space(60%), fusion of the vertebra (40%), restricted spinal movement and local kyphosis(20%), residual defects (20%)	Antibiotics and immobilization (a plaster bed or cast or brace) (100%) for 16–80 weeks, no surgical management
Karabouta Z., et al. 2005	Reduced ability or inability to walk or sit (100%), back pain (100%), abdominal pain (60%), limp (20%), reduced range of movement (20%), fever (20%)	Lumbar spine (100%)	4 weeks	Normal ESR, CRP, and blood counts	Negative blood cultures (100%)	X-ray: disc space narrowing and irregular end plates of vertebrae (80%) MRI: abnormal disc signal (100%), paravertebral mass (20%)	3 months follow –up: no evidence of complications	Antibiotic (100%), no surgical management
Garron E.,et al. 2002	Fever(60%), back pain (50%), limp (38%), reduced ability or inability to walk or sit (21%), abdominal pain (10%), neurological signs (10%)	Cervical spine (7%), thoracic spine (21.4%), lumbar spine (71.6%)	6weeks	ESR elevation (71%), CRP elevation (50%), leukocytosis (50%)	Positive blood culture and biopsies: *C.burnetii* (3cases) FNA: *S.aureus* (55%), *K.kingae* (27%)	X-rays: narrowing of intervertebral disk and destruction of adjacent vertebral endplate (100%) MRI and CT scan: perispinal abscess (37%), epidural thickening (19%) Bone scan: increase uptake at effected disk and adjacent vertebral bodies (61%)	52.8 months follow-up: kyphosis (16%), pain in activities (7%), limited neck mobility (2%), neural sequelae (2%),	Antibiotics and immobilization (collar and brace) (100%), surgical intervention (9.5%)
Brown.R.,et al.2001	Reduced ability or inability to walk or sit (63%), inability to flex the lower back(50%), loss of lordosis (40%), back pain (27%), neurological signs(9%)	Lumbar spine (100%)	3.4weeks	ESR elevation (100%), leukocytosis (64%), CRP elevation (40%),	Negative blood cultures (100%), Biopsy: inflammatory cells (18%)	X-rays: narrowing of intervertebral disk (55%), MRI: reduced disk height, abnormal disc signal (72%), destruction of endplates or protrusion of the disc (55%), paraspinal mass (27%)	34 months follow up: kyphosis (9%), osseous fusion (18%), posterior wedging (9%)	Antibiotics (100%) for 2–42 weeks, immobilization (45%) (brace) for 11 weeks, no surgical management

*C.burnetii :Coxiella burnetii, S.aureus: Staphylococcus aureus, K.kingae: Kingella kingae, S.epidermis: Staphylococcus epidermidis, S.pneumonia: Streptococcus pneumonia, K.pneumonia: Klebsiella pneumonia, M.tuberculosis: Mycobacterium tuberculosis, S.typhi: Salmonella typhi, L.lactis: Lactococcus lactis, S.agalactiae: Streptococcus agalactia, MSSA:Methicillin-resistant Staphylococcus aureus, M.morganaii:Morganella morganii*

ESR: Erythrocyte sedimentation rate, CRP: C-reactive protein, MRI: magnetic resonance imaging, CT scan: computerized tomography scan, N.A.: not avaliable

The mean delay in the time of diagnosis was 4.8 weeks, and this delay, according to different studies, varies from 2 days to 60 weeks [6–22].

Clinical manifestations of discitis in children are nonspecific. Most studies (15 studies) (88%) reported back pain [6, 7, 9, 11–22] and fever [6, 8–18, 20–22] as the most common causes of complaints, followed by eleven studies reporting reduced ability or inability to walk or sit [6, 7, 9, 11–16, 20, 21]. Other commonly observed findings include limping, reduced range of movements, abdominal pain, reduced general condition, neurological signs, torticollis, and extra-skeletal signs [6–22].

The most affected site of all the studies was the lumbar spine ( the most affected segments are L3-L4 and L5-S1) [6–22]; four studies just reported on the lumbar spine [7, 8, 14, 19]. Therefore, the thoracic is in second place (12 studies) [6, 9–13, 15, 17, 18, 20–22], and the cervical is in third place (9 studies) [6, 10–13, 15, 17–19]. Only five studies have addressed sacral spine involvement [10, 11, 13, 17, 18].

### Laboratory investigation and microbiological findings

Most studies (94%) reported an increase in inflammatory markers such as white blood cell count (WBC) (> 11 × 10^3^/micL), C-reactive protein (CRP), and erythrocyte sedimentation rate (ESR). Elevated ESR (> 20 mm/h) was reported in 16 studies [6–13, 15–22], and one reported as normal [14]. Fourteen studies reported elevated CRP (> 5 mg/L) [6–13, 16–19, 21, 22] and eleven studies reported leukocytosis [6, 7, 9–13, 16, 17, 19, 22]. One study reported anemia [10] and two studies reported thrombocytosis [8, 10]. One study reported normal CRP and white blood cells [14].

In sixteen studies (94%), blood culture was taken from patients. Negative blood cultures were reported in six studies [7, 8, 14, 15, 20, 21], whereas positive blood cultures were reported in ten studies [6, 9–13, 17–19, 22].

In seven studies (41%), they performed fine needle aspiration and took biopsies, which were reported positive [6, 7, 10–13, 21]. Less than 30% of patients had both positive blood cultures and biopsy findings.

The most common microbiological findings (64%) were *Staphylococcus Aureus* and *Kingella kingae* [6, 9–13, 16–19, 22]. Other findings were less than 5% of patients, including *Mycobacterium tuberculosis, Coxiella burnetii, E. coli, Streptococcus agalactiae, Staph epidermis, Streptococcus pneumonia, and Klebsiella pneumonia* [6, 9, 10, 12–14, 17–19, 22].

In the studies conducted by Yagdiran A. et al. (2022) and Roversi M. et al. (2021), it was observed that granulomatous spondylodiscitis (*Mycobacterium tuberculosis*) was found in the patients who participated in the studies [18, 21].

### Radiographic findings

In radiographic evaluation, X-rays and MRI with injection are useful, and MRI is the study of choice for diagnosing of SD. Fifteen studies examined X-ray findings, and in most cases, the intervertebral disk’s narrowing, lumbar lordosis reduction, loss of disk height, destruction of the vertebral body, and irregularity of the vertebral erosions of adjacent vertebral endplate have been reported [6, 7, 9–21].

In 15 studies, MRI reported reduced disk height, disk hypointensity on T1, disk hyperintensity in T2, destruction of the adjacent vertebral endplate, epidural collection, Cellulitis or myositis, and inflammatory mass [6–14, 16–21].

Four studies showed that CT detected inflammatory masses and abscesses [12, 13, 17, 18]. Four studies reported bone scans of increased resorption in the affected disc and adjacent vertebral bodies [9, 12, 15, 19].

### Treatment and follow-up

All the studies initiated antibiotic therapy [6–22], and nine studies used immobilization in addition to treatment [7, 9, 11, 12, 15, 16, 18, 20, 22]. In 5 studies, surgery was performed for patients due to clinical indications [11–13, 17, 21]. The follow-up period was varied in studies, and it differed from 1.5 months to 156 months. The variation in follow-up periods was related to the role of age, complications, and pathogens [6–9, 11–22].

### Complications

The complications are related to age, delay in treatment, pathogen, and how they were managed. Sixteen studies reported complications and the most common complications are vertebral body destruction, muscle abscesses, back pain, kyphosis, reduced range of movement, scoliosis, neurological complications, fibrous ankyloses, high-grade narrowing of the intervertebral disc space, and fusion of the vertebra [6–9, 11–22].

## Discussion

Spondylodiscitis is an infrequent disease involving disc spaces, adjacent discs, and vertebral bodies. It remains a rare diagnosis, but its incidence has increased in recent years due to more effective diagnostic methods allowing for earlier diagnosis. Although affected sex varies between studies, most studies show that boys appeared to be more affected than girls. This disease can affect any age; most patients are between 2 and 8 years old. In the studies, patients were reported from 24 days to 17.5 years old, but the average age was 4.3 years [6–22].

According to these studies, clinical features vary by age group and are nonspecific. Neonates or infants often have severe spondylodiscitis, frequently associated with sepsis and infectious diseases. However, spondylodiscitis signs and symptoms in toddlers and preschool children are usually mild. Infants under one year cannot express symptoms. Their parents often refer them for nonspecific symptoms such as hypotension, refusing to bear weight, fever, and general illness.This issue leads to delayed and inaccurate diagnoses for these patients. The most frequently reported symptom in preschool children was back pain, which was pre-existing and localized to the part of the spine affected by the infection. This pain is accompanied by limping, stiffness, and a reduced range of motion. Movement often aggravates it and can spread to other organs (abdomen, hips, legs, and scrotum). Symptoms commonly reported in toddlers are irritability, limping, reduced ability or inability to walk or sit, and poor general condition. The most frequent signs at the physical examination were fever, torticollis, inability to flex the lower back, antalgic gait, limitation of spinal movements (passive or active), and tenderness or swelling at palpation. There were also extra-skeletal manifestations, such as abdominal pain, constipation, and neurological signs (spinal cord or nerve root compression and meningitis). A few studies mentioned abdominal pain as a significant symptom of spondylodiscitis. In a survey conducted by Garron E. et al. (2002), they declared that although abdominal pain is irrelevant to the nature of spondylodiscitis, it appears this pain was a result of retroperitoneal irritation of the psoas abscess in these patients. Also, abdominal pain in these children can mask the typical symptoms of spondylodiscitis in patients, leading to a delay in diagnosis and, in some cases, misdiagnosis [1, 2, 4, 12, 27–31].

The lumbar spine is the most common level of involvement, especially in children under five years old. The most affected segments are L3-L4 and L5-S1. The lumbar spine seems prone to spondylodiscitis due to disruption of venous drainage through the Batson plexus. In a study by Musso P. et al. (2021), the incidence of involvement of the lumbar or lumbosacral region represents the majority of cases (75% of patients) [6–10, 17, 28, 30, 31].

Because the initial clinical manifestations are nonspecific, discitis diagnosis is usually made relatively late in the disease process. Several studies have described a delay of 4 to 6 weeks. This delay can lead to significant complications compared to an early diagnosis. Also, nonspecific manifestations in non-verbal infants lead to delayed diagnosis. In the study by Afshari F. et al. (2019), the median duration of symptoms before onset was six weeks, reflecting the nonspecific nature of the disease and the difficulty of diagnosing it in children. A significant delay in treatment can destroy the disc space, epidural fluid collection, nerve compression, and deformity [6, 10, 18–22, 28, 29].

The diagnosis of spondylodiscitis is based on clinical findings, a radiographic survey, laboratory evaluation, and, in some cases, FNA results. Elevation of inflammatory markers is typical in most cases; however, it is not specific. These findings are clinically useful indicators and should be used to diagnose and monitor disease progression and treatment follow-ups. Most studies showed patients had ESR levels ≥ 20 mm/h and CRP ≥ 10 mg/L on admission day. The highest values of inflammatory markers are usually presented in younger patients, those with severe diseases, patients with poor general conditions, and those with complicated diseases. Therefore, these markers seem reliable for identifying spondylodiscitis and following up on disease outcomes. According to the Roversi M. et al. (2021) study, the ESR test has more sensitivity than WBC and CRP and has increased in most infectious cases. In order to check and follow up on the treatment with the help of inflammatory markers, the tests should be repeated as follows: WBC, CRP, and ESR start every second day, then after resolving the fever, twice per week and once every two weeks. When the diagnosis is delayed, there is a higher probability of high inflammatory markers. On the other hand, when patients have received antibiotic medication in outpatient treatment, the likelihood of positive inflammatory markers is less. Elevation of ESR can also be detected in viral infection or inflammatory processes, which can be a sign of other etiologies of spondylodiscitis rather than bacterial infection, especially in cases with isolated elevated ESR [6–13, 15–22].

The specific etiology of spondylodiscitis is still unclear; however, as stated in the studies, infectious diseases are believed to be a common cause. Therefore, blood cultures and biopsies were performed to investigate the cause of infection in these patients [31–36].

Blood cultures can be positive in less than 30% of patients and help guide antibiotic therapy choices. Attempts should always be made to obtain direct cultures of the vertebral body and intervertebral disc space if less invasive culture techniques fail to identify the organism. Cultures of bacteria, fungi, and mycobacteria should also be performed in cases with nonspecific manifestations and fever. According to the studies, they suggested antibiotic therapy be withheld until cultures are obtained if it is clinically possible. Kayser R,. et al.2005, reported that one of the causes of negative results of blood cultures is antibiotic therapy of patients before hospitalization [6, 9–13, 17–19, 22].

Furthermore, some patients have been treated with antibiotics due to a history of other diseases or infections, which has caused negative culture results. However, not all patients who had a negative blood culture were previously treated with antibiotics or had a history of previous diseases. This difference can be due to other etiologies of spondylodiscitis besides bacterial infectious diseases, including viral infections or inflammatory conditions, or, in some cases, the possibility of a failure of the organism growing in the culture, errors in sampling techniques, and the unavailability of tissue samples [6–22, 31–34].

A biopsy is not necessary for patients demonstrating clear clinical features but is indicated for those whose symptoms do not rapidly improve with treatment or who have atypical presentations. Due to low yield, morbidity, and the need for sedation or general anesthesia in young children, a biopsy is not routinely recommended to evaluate children with spondylodiscitis. Biopsy results often yield low results due to confounding factors such as inadequate sampling, culture technique, previous antibiotic treatment, or rapid host response to a low-virulence pathogen [12, 14, 18, 29–35].

Spondylodiscitis has four radiographic stages: the latent stage, when x-rays are frequently normal; the acute phase, which occurs within two to four weeks of the onset of symptoms and is characterized by narrowing and erosion of the disc space; the healing phase, which takes place two to three months after radiography changes and is characterized by sclerosis of the contours of the vertebral body; and late stage, is characterized by a narrowing of the affected disc space [6–21, 29–31].

The initial imaging study should be plain radiographs of the suspected area to rule out other causes of pain. However, radiographs at the onset of discitis are usually normal and, after 2 or 3 weeks, show the disease’s progression and often require other advanced techniques. The primary X-ray features suggest loss of disc height, periosteal abscess, bone consolidation, destruction of the vertebral body, irregularity of the vertebral erosions of the adjacent vertebral endplate, and intervertebral disc space narrowing. Plain radiographs reveal isolated intervertebral disk space narrowing when symptoms present for one week or more. By 3 to 4 weeks, tooth erosion of adjacent vertebral endplates had been seen [6–21, 29–37].

Technetium Tc 99 m (bone scan) can isolate the area of pathology to a specific motion segment and help confirm the diagnosis if plain X-rays are unaltered. A bone scan benefits children whose localization of the infected area can be very difficult only by physical examination. After the onset of clinical symptoms, changes may appear as early as 3–5 days [9, 12, 15, 19].

CT scans are more sensitive and valuable, especially for studying areas that are difficult to scan on X-rays, such as the dorsolumbar or lower cervical spine. In half of cases, CT abnormalities are visible in the first two weeks of infection. The evaluation of markers will look for early disc involvement presenting as reduced bone density. Areas of osteolysis, bone erosion, or vertebral endplate geodes can be easily identified, and this method helps to visualize bone sequestra within the canals, residual calcification, and the presence of gas within an abscess [12, 13, 17, 18, 38–40].

MRI is the gold standard method for diagnosing spondylodiscitis and has higher sensitivity and specificity than other methods. MRI is valuable because it provides an excellent image visualization of the disk, neural tissues, surrounding soft tissues, and pathophysiologic changes in the vertebral body. Hence, this can reduce diagnostic delay while guiding the extent and duration of treatment. MRI findings are high signal intensity from the discs and the two adjacent vertebrae, thickening of the paravertebral soft tissue, involvement within the vertebral canal on T2-weighted images, and low signal intensity on T1. The patient’s age must be considered because red bone marrow remains predominant in children, and the high signal intensity on T1 could mask the inflammation [6–14, 16–21].

One of the considerable challenges is choosing the best protocol for treatment. Unfortunately, until today, the lack of a coherent practical guideline in this regard has led doctors to select the appropriate treatment based on their clinical judgment and the patient’s condition. The goal of treatment is to eliminate the infection and minimize morbidity. Different responses to different treatment regimens for spondylodiscitis have led to ongoing confusion and discussion about its underlying cause.

Most studies report that intravenous antibiotics are recommended as initial management, and delaying antibiotic therapy is associated with prolonged hospitalization, recurrent symptoms, worsening infections, and complicated conditions. The antibiotic treatment is recommended for patients with pain, scoliosis, persistently inflammatory markers, high fever, extreme pain, or positive blood, sputum, throat, or urine cultures. Empirical treatment focuses on S. aureus, the most common organism isolated from culture-positive biopsy specimens. There are no absolute recommendations regarding the duration of antibiotic treatment. Initial treatment is with parenteral antibiotics (range 1–8 weeks), and if there is clinical and experimental evidence of patient response to antibiotic therapy, the antibiotic therapy continues with oral antibiotics (range 3–6 months). Yagdiran A. et al. (2022) announced that because the most common cause of infection in spondylodiscitis is Staphylococcus aureus and it takes time to get the blood culture result, physicians start the empirical treatment based on the antibiotic covering for Staphylococcus aureus after taking the culture sample. Antibiotic treatment has been different in each region, and it is prescribed based on the disease’s severity and the child’s age; conversely, there is no specific guideline for treating spondylodiscitis in patients. Hence, doctors start the treatment based on common infection pathogens and bacterial resistance in their region [6–22, 29, 31–39, 41, 42].

Immobilization using bed rest, a cast, or a brace has usually been recommended, and it decreases pain and minimizes deformity in patients. Relative rest and immobilization can be used along with antibiotics to improve spondylodiscitis. In a study by Chandrasenan J. et al. (2011), they did not prescribe antibiotics to patients without infectious clinical symptoms who had negative blood cultures and normal white blood cell counts. They were treated only with bracing, recovered completely, and had no problems in the follow-up periods. They recommend spinal bracing without antibiotics when children are systemically well with a normal WBC count and a negative blood culture [7, 9, 11, 12, 15, 16, 18, 20, 22].

Surgical debridement should only be considered in rare patients with documented abscesses with a systemic disease or ongoing neurological deficits, significant destruction of the vertebral bodies, progressing to rapid transformation of the deformity into kyphosis, spinal instability, or failure of conservative treatment [11–13, 17, 21].

After symptoms resolved with antibiotic treatment, patients were followed for 12 to 18 months. Recurrence of symptoms, although rare after treatment, should prompt the treating physician for a new x-ray, complete blood count, C-reactive protein level, and ESR. The study by Frerri et al. (2021) suggested that a long-term follow-up is necessary for children to compare the effectiveness of treatment because many aspects of the treatment are still under debate [11, 31–37].

Although there is no definitive indication for surgical procedures, surgery treatment is advised for patients with compression of neural elements, progressive neurologic impairment, mechanical derangement (instability, malalignment, and severe bone destruction), intractable pain, and evacuation abscesses. Zomalheto Z. et al. (2013) declared that most children diagnosed with neurological symptoms were more likely to develop neurological complications after treatment. They also mentioned that patients with initial nonspecific symptoms had a higher prevalence of complications due to delays in diagnosis [12–22].

Our review is comprehensive and includes both cohort and cross-sectional studies. This study included all studies evaluating this condition in children. The majority of studies were of good quality. Children’s discitis is a relatively rare condition; therefore, few studies have been conducted. The studies were retrospective, there were no predefined management protocols, and there was a lack of valuable data, especially regarding treatment rationale in some children. It is impossible to homogenize and meta-analyze the data due to the small number of studies and the heterogeneity of study design and results. Most studies have been conducted on a limited number of patients for a short time. These studies should involve more patients and a more prolonged treatment and follow-up period. Age-matched control groups are also recommended for these studies.

## Conclusion

Clinical manifestations vary with the child’s age, but characteristic findings on physical examination, laboratory tests, radiographs, and bone scans allow early diagnosis. Early diagnosis can prevent unnecessary tests and invasive procedures. Empirically-directed intravenous antibiotic therapy for S. aureus is the mainstay of treatment. Immobilization and rest can be added to relieve symptoms. Follow-up should continue for 12–18 months after healing. Biopsy and surgery are reserved for patients unresponsive to intravenous antibiotic therapy.

### Electronic supplementary material

Below is the link to the electronic supplementary material.

Supplementary Material 1

Supplementary Material 2

## Data Availability

All data generated or analysed during this study are included in this published article [and its supplementary information files].

## References

[1] Gentile L, Benazzo F, De Rosa F, Boriani S, Dallagiacoma G, Franceschetti G, Gaeta M, Cuzzocrea F (2019). A systematic review: characteristics, Complications and treatment of spondylodiscitis. Eur Rev Med Pharmacol Sci.

[2] Taylor DG, Buchholz AL, Sure DR, Buell TJ, Nguyen JH, Chen CJ, Diamond JM, Washburn PA, Harrop J, Shaffrey CI, Smith JS (2018). Presentation and outcomes after medical and surgical treatment versus medical treatment alone of spontaneous infectious spondylodiscitis: a systematic literature review and meta-analysis. Global Spine Journal.

[3] Saleh ES, Vasileff CC, Omari AM, Khalil JG, Omari A, Khalil J (2021). The diagnosis and management of pediatric spine Infections. Cureus.

[4] Principi N, Esposito S (2016). Infectious discitis and spondylodiscitis in children. Int J Mol Sci.

[5] de Fucs MB, Meves PM, Yamada R (2012). Spinal Infections in children: a review. Int Orthop.

[6] Afshari FT, Rodrigues D, Bhat M, Solanki GA, Walsh AR, Lo WB (2020). Paediatric spondylodiscitis: a 10-year single institution experience in management and clinical outcomes. Child’s Nerv Syst.

[7] Brown R, Hussain M, McHugh K, Novelli V, Jones D (2001). Discitis in young children. J Bone Joint Surg Br Vol.

[8] Ceroni D, Belaieff W, Kanavaki A, Della Llana RA, Lascombes P, Dubois-Ferriere V, Dayer R. Possible association of Kingella kingae with infantile spondylodiscitis. The Pediatric infectious disease journal., Ceroni D, Belaieff W, Kanavaki A, Della Llana RA, Lascombes P, Dubois-Ferriere V, Dayer R. Possible association of Kingella kingae with infantile spondylodiscitis. The Pediatric infectious disease journal. 2013;32(11):1296-8. 10.1097/INF.0b013e3182a6df50 24131988

[9] Chandrasenan J, Klezl Z, Bommireddy R, Calthorpe D (2011). Spondylodiscitis in children: a retrospective series. J Bone Joint Surg Br Vol.

[10] Dayer R, Alzahrani MM, Saran N, Ouellet JA, Journeau P, Tabard-Fougère A, Martinez-Álvarez S, Ceroni D (2018). Spinal Infections in children: a multicentre retrospective study. Bone Joint J.

[11] Ferri I, Musso P, Ristori G, Galli L, Chiappini E. Epidemiology and Management of Pyogenic Spondylodiscitis in a Tertiary Paediatric Center, over 10 Years.

[12] Garron E, Viehweger E, Launay F, Guillaume JM, Jouve JL, Bollini G (2002). Nontuberculous spondylodiscitis in children. J Pediatr Orthop.

[13] Kang HM, Choi EH, Lee HJ, Yun KW, Lee CK, Cho TJ, Cheon JE, Lee H (2016). The etiology, clinical presentation and long-term outcome of spondylodiscitis in children. Pediatr Infect Dis J.

[14] Karabouta Z, Bisbinas I, Davidson A, Goldsworthy LL (2005). Discitis in toddlers: a case series and review. Acta Paediatr.

[15] Kayser R, Mahlfeld K, Greulich M, Grasshoff H (2005). Spondylodiscitis in childhood: results of a long-term study. Spine.

[16] Miranda I, Salom M, Burguet S (2014). Discitis in children less than 3 years: a case series and literature review. Revista Española De Cirugía Ortopédica Y Traumatología. (English Edition).

[17] Musso P, Parigi S, Bossi G, Marseglia GL, Galli L, Chiappini E (2021). Epidemiology and management of acute hematogenous osteomyelitis, neonatal osteomyelitis and spondylodiscitis in a third level paediatric center. Children.

[18] Roversi M, Mirra G, Musolino A, Barbuti D, Lancella L, Deriu D, Iorio C, Villani A, Crostelli M, Mazza O, Krzysztofiak A (2021). Spondylodiscitis in children: a retrospective study and comparison with non-vertebral osteomyelitis. Front Pead.

[19] Spencer SJ, Wilson NI (2012). Childhood discitis in a regional children’s hospital. J Pediatr Orthop B.

[20] Waizy H, Heckel M, Seller K, Schroten H, Wild A (2007). Remodeling of the spine in spondylodiscitis of children at the age of 3 years or younger. Arch Orthop Trauma Surg.

[21] Yagdiran A, Meyer-Schwickerath C, Wolpers R, Otto-Lambertz C, Mehler K, Oberthür A, Kernich N, Eysel P, Jung N, Zarghooni K (2022). What do we know about Spondylodiscitis in Children? A retrospective study. Children.

[22] Zomalheto Z, Assogba M, Zannou V (2018). Prevalence and risk factors of infectious spondylodiscitis in Benin’s children. Open J Rheumatol Autoimmune Dis.

[23] Lashkarbolouk N, Mazandarani M, Azari AA, Shahkar L, Shariatalavi R. Neuroblastoma in early age with rare respiratory manifestation. J Compr Pediatr. 2022;13(4).

[24] Sharififar R, Heidari K, Mazandarani M, Lashkarbolouk N. Comparison of the polymerase chain reaction method with serological tests in the diagnosis of human brucellosis. Jundishapur J Microbiol. 2023;16(2).

[25] Liberati A, Altman DG, Tetzlaff J, Mulrow C, Gøtzsche PC, Ioannidis JP (2009). The PRISMA statement for reporting systematic reviews and meta-analyses of studies that evaluate health care interventions: explanation and elaboration. PLoS Med.

[26] Moskalewicz A, Oremus M (2020). No clear choice between Newcastle–Ottawa Scale and Appraisal Tool for cross-sectional studies to assess methodological quality in cross-sectional studies of health-related quality of life and Breast cancer. J Clin Epidemiol.

[27] Wells G, Shea B, O’Connell D, Peterson J, Welch V, Losos M, et al. Newcastle-Ottawa quality assessment scale cohort studies. University of Ottawa; 2014.

[28] Leone A, Dell’Atti C, Magarelli N, Colelli P, Balanika A, Casale R, Bonomo L (2012). Imaging of spondylodiscitis. Eur Rev Med Pharmacol Sci.

[29] Alanay A, Yilgor C. Acute Pediatric Spinal Infections. The Growing Spine: Management of Spinal Disorders in Young Children. 2016:473 – 81.

[30] Petkova AS, Zhelyazkov CB, Kitov BD (2017). Spontaneous spondylodiscitis-epidemiology, clinical features, diagnosis and treatment. Folia Med.

[31] Tyagi R (2016). Spinal Infections in children: a review. J Orthop.

[32] Raghavan M, Lazzeri E, Palestro CJ. Imaging of spondylodiscitis. InSeminars in Nuclear Medicine 2018 Mar 1 (Vol. 48, No. 2, pp. 131–47). WB Saunders. 10.1053/j.semnuclmed.2017.11.001 29452617

[33] Rutges JP, Kempen DH, Van Dijk M, Oner FC (2016). Outcome of Conservative and surgical treatment of pyogenic spondylodiscitis: a systematic literature review. Eur Spine J.

[34] Machado SA, Freitas JM, da Silva NP, Moreira JM, Pinto RA, de Melo Costa FG (2017). Spondylodiscitis by Kingella kingae: an emerging pathogen in an older pediatric population. Pediatr Infect Dis J.

[35] Autore G, Bernardi L, Esposito S (2020). Update on acute bone and joint Infections in paediatrics: a narrative review on the most recent evidence-based recommendations and appropriate antinfective therapy. Antibiotics.

[36] McNamara AL, Dickerson EC, Gomez-Hassan DM, Cinti SK, Srinivasan A (2017). Yield of image-guided needle biopsy for infectious discitis: a systematic review and meta-analysis. Am J Neuroradiol.

[37] Ferri I, Ristori G, Lisi C, Galli L, Chiappini E (2020). Characteristics, management and outcomes of spondylodiscitis in children: a systematic review. Antibiotics.

[38] Gallina P, Dardo M, Pedone A, Travaglini F (2023). Clinical image: spondylodiscitis as a complication of urosepsis caused by extracorporeal shock wave lithotripsy for kidney stones. Oxf Med Case Rep.

[39] Al Yazidi LS, Hameed H, Kesson A, Isaacs D (2022). Spondylodiscitis in children. J Paediatr Child Health.

[40] Bianchini S, Esposito A, Principi N, Esposito S. Spondylodiscitis in paediatric patients: the importance of early diagnosis and prolonged therapy. International journal of environmental research and public health. 2018;15(6):1195.neu. 10.3390/ijerph15061195 PMC6025454 29875345

[41] Gregori F, Grasso G, Iaiani G, Marotta N, Torregrossa F, Landi A (2019). Treatment algorithm for spontaneous spinal Infections: a review of the literature. J Craniovertebral Junction Spine.

[42] Yagupsky P, Ceroni D. An update on pediatric skeletal system Infections. Front Pead. 2023;11. 10.3389/fped.2023.1128126 PMC9969156 36861071

