# Supplementary material for: Understanding the management of pediatric spondylodiscitis based on existing literature; a systematic review
Source: BMC Pediatr. 2023 Nov 18;23:578. doi: 10.1186/s12887-023-04395-2 (PMC10656982; doi:10.1186/s12887-023-04395-2)
Supplement: Supplementary file 1 — Supplementary Material 1 [file 12887_2023_4395_MOESM1_ESM.docx]

**Supplementary Table 1. Newcastle-Ottawa Scale Adapted for Retrospective Cohort Studies**

| First author, (Year) | Study design | Selection | | | | Comparability | Outcomes | | | Quality score |
| --- | --- | --- | --- | --- | --- | --- | --- | --- | --- | --- |
|  |  | Representativeness of the exposed | Selection of the non-exposed | Ascertainment of exposurents | Demonstration that outcome |  | Assessment | Follow-up | Median duration of follow-up |  |
| **Yagdiran A.,et al.2022** | Cohort study | 1 | 0 | 1 | 1 | 2 | 1 | 1 | 1 | Good |
| **Ferri I.,et al.2021** | Cohort study | 1 | 0 | 1 | 1 | 2 | 1 | 1 | 1 | Good |
| **Musso P.,et al.2021** | Cohort study | 1 | 0 | 1 | 1 | 1 | 1 | 1 | 1 | Good |
| **Roversi.M.,et al.2021** | Cohort study | 1 | 0 | 1 | 1 | 1 | 1 | 1 | 1 | Good |
| **Afshari F.,et al.2019** | Cohort study | 1 | 0 | 1 | 1 | 2 | 1 | 1 | 1 | Good |
| **Dayer R.,et al. 2018** | Cohort study | 1 | 0 | 1 | 1 | 2 | 1 | 0 | 0 | Poor |
| **Kang H.,et al.2016** | Cohort study | 1 | 0 | 1 | 1 | 2 | 1 | 1 | 1 | Good |
| **Ceroni D.,et al.2013** | Cohort study | 1 | 0 | 1 | 1 | 2 | 1 | 1 | 1 | Good |
| **Spencer S.,et al.2012** | Cohort study | 1 | 0 | 1 | 1 | 1 | 1 | 1 | 1 | Good |
| **Chandrasenan J.,et al. 2011** | Cohort study | 1 | 0 | 1 | 1 | 2 | 1 | 1 | 1 | Good |
| **Miranda.I.,et al.2008** | Cohort study | 1 | 0 | 1 | 1 | 2 | 1 | 1 | 1 | Good |
| **Waizy H.,et al.2007** | Cohort study | 1 | 0 | 1 | 1 | 2 | 1 | 1 | 1 | Good |
| **Kayser R.,et al.2005** | Cohort study | 1 | 0 | 1 | 1 | 2 | 1 | 1 | 1 | Good |
| **Karabouta Z.,et al.2005** | Cohort study | 1 | 0 | 1 | 1 | 1 | 1 | 1 | 1 | Good |
| **Garron E.,et al. 2002** | Cohort study | 1 | 0 | 1 | 1 | 2 | 1 | 1 | 1 | Good |
| **Brown.R.,et al.2001** | Cohort study | 1 | 0 | 1 | 1 | 1 | 1 | 1 | 1 | Good |
