# Supplementary material for: Understanding the management of pediatric spondylodiscitis based on existing literature; a systematic review
Source: BMC Pediatr. 2023 Nov 18;23:578. doi: 10.1186/s12887-023-04395-2 (PMC10656982; doi:10.1186/s12887-023-04395-2)
Supplement: Supplementary file 2 — Supplementary Material 2 [file 12887_2023_4395_MOESM2_ESM.docx]

**Supplementary Table 2. Newcastle-Ottawa Scale Adapted for Cross Sectional Studies**

| **Study** | **Selection** | | | |  | **Compatibility** | |  | **Outcome** | | **Total** |
| --- | --- | --- | --- | --- | --- | --- | --- | --- | --- | --- | --- |
|  | **Representativeness of cases** | **Sample size** | **Non-respondents** | **Ascertainment of the exposure** |  | **On age** | **On other risk factors** |  | **Assessment of the outcome** | **Statistical test** |  |
| Zomalheto Z.,et al.2013 | 1 | 1 | 1 | 0 |  | 1 | 1 |  | 2 | 0 | Good |
